# The Eye of the Beholder: Male Mate Choice Is Influenced by Partner Infection Status and Self‐Condition

**DOI:** 10.1002/ece3.72178

**Published:** 2025-09-27

**Authors:** Carolina Castillo, Jimena Meneses Placencia, Judith Ulloa, Sagrario Cordero‐Molina, Constantino Macías García, Indrikis Krams, Jorge Contreras‐Garduño

**Affiliations:** ^1^ Laboratorio de Ecología Evolutiva, Escuela Nacional de Estudios Superiores Universidad Nacional Autónoma de México Morelia Michoacan Mexico; ^2^ Instituto de Ecología Universidad Nacional Autónoma de México Mexico City Mexico; ^3^ Institute of Ecology and Earth Sciences University of Tartu Tartu Estonia; ^4^ Department of Ecology, Faculty of Medicine and Life Sciences University of Latvia Riga Latvia; ^5^ Department of Biodiversity Institute of Life Sciences and Technology, Daugavpils University Daugavpils Latvia

## Abstract

Research on mate choice has predominantly emphasized the female perspective, often neglecting the male viewpoint. In studies where infection status is considered a factor in mate choice, two critical questions emerge: (a) how does the condition of the choosing individual influence mate preferences? and (b) do the choosier sex prioritize healthy partners because of their attractiveness, or to mitigate infection risk? Here we investigated these questions in *Tenebrio molitor* male mate preferences concerning male condition and female infection status. We found that males preferred healthy females over infected ones, which corresponded with an increased likelihood of egg‐laying by healthy females. Importantly, survival rates did not differ between males mating with infected versus healthy females. Notably, when males were food‐deprived, they showed no preference between infected and control females. This lack of preference was not attributable to infection avoidance, as males did not distinguish between (a) infected and healthy males, or (b) infected and sterilized food sources. These results support the condition‐dependent preference hypothesis, suggesting that male mate choice is primarily influenced by their own condition rather than by parasite avoidance.

## Graphical abstract

Research on mate choice has traditionally emphasized the female perspective, with relatively less attention given to male choice. Here, we demonstrate that (a) males preferentially chose healthy females due to their higher attractiveness rather than to avoid infection risk, and (b) this preference was modulated by the males' own condition.

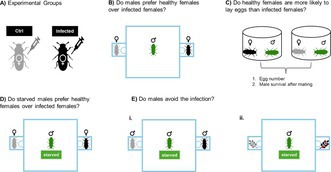

## 1 Introduction

Parasitic load plays a vital role in host reproductive strategies, as its presence can negatively impact the development of secondary sexual characteristics (SSC) and serve as an honest signal of genetic quality and overall condition to the choosy sex (Hamilton and Zuk 1982). The threat posed by parasites compels hosts to navigate a trade‐off between allocating resources to immune defenses and reproduction. Consequently, only high‐quality individuals are capable of effectively balancing this trade‐off, thereby exhibiting honest signals of parasite resistance that are likely to be favored by mate choice and transmitted to future generations (Zahavi 1977; Hamilton and Zuk 1982). Under stressful environmental conditions, such as nutritional deficits or infections, cellular function may be compromised, leading to a decline in overall organismal condition (Hill 2011). As a result, individual conditions may influence not only mate choice (specifically, which sex is chosen) but also the mating decisions made by the choosing sex.

Traditional research has primarily emphasized female preference for high‐quality males capable of resisting parasites while maintaining costly SSC (Zahavi 1977; Hamilton and Zuk 1982). However, male mate choice under certain ecological and evolutionary contexts, such as female infection status, has received comparatively less attention (Bonduriansky 2001; Edward and Chapman 2011). Parasites often diminish female reproductive success, as evidenced by impaired fertility (Wittman and Fedorka 2015), modifications in sexual traits such as coloration (Daspre et al. 2009; Paciência et al. 2019), and alterations in pheromone production (Gonzalez‐Karlsson et al. 2021). These factors may, in turn, influence male mate choice (Bonduriansky 2001; Edward and Chapman 2011). Nonetheless, the extent to which males actively avoid parasitized females or detect and reject them based on honest signals of health remains an area that is still insufficiently explored.

We hypothesize that parasitized females reduce their attractiveness due to weakened quality signals, thereby eliciting male preference for healthy females. To evaluate this hypothesis, we used *Tenebrio molitor* , a gregarious and polygamous species that relies on chemical cues for mate attraction (August 1971; Bryning et al. 2005; Tanaka et al. 1986; Nielsen and Holman 2012). Female mate choice in this species has been extensively studied, with evidence showing that females prefer males producing higher amounts of the aggregation pheromone (Z)‐3‐dodecenyl acetate (Z3‐12:Ac) (Bryning et al. 2005). Production of this pheromone, and thus male condition, is known to decline under oxidative stress (Ruiz‐Guzmán et al. 2021), starvation (Rantala et al. 2002), and parasitic infection (Worden et al. 2000; Nielsen and Holman 2012). In contrast, studies on male mate choice indicate that males generally prefer females in good condition, which produce greater amounts of 4‐methyl‐1‐nonanol (Tanaka et al. 1986), compared to females in poorer condition due to reproductive status (Carazo et al. 2004), age (Vanderwel et al. 2017), or oxidative stress (Ruiz‐Guzmán et al. 2021). Nonetheless, current evidence suggests that males may be unable to discriminate between healthy and parasitized females (Gao et al. 2021). This gap leaves the question of how males respond to parasitized females insufficiently addressed.

The nature of the parasitic challenge is a key factor in understanding how parasites influence mate choice, because this may shape mating strategies under parasitic pressure (Beltran‐Bech and Richard 2014). Based on this framework, we hypothesized that infection in *T. molitor* females by the entomopathogenic fungus *Metarhizium anisopliae* (Joop and Vilcinskas 2016) would reduce the female attractiveness to males. Specifically, we predicted that males would preferentially associate with healthy females over infected females. In addition, we hypothesized that mating with parasitized females would impose reproductive costs on males, reflected in a reduced number of eggs laid by infected compared to healthy females. Another factor known to influence mate choice is resource availability, as it can modify reproductive decisions (Krams et al. 2015). For instance, in beetles, healthy females or those in poor condition (e.g., experiencing oxidative stress) tend to prefer healthy partners, whereas males under the same conditions are less selective and show no consistent preference between healthy and stressed females (Ruiz‐Guzmán et al. 2021). Similarly, in marine iguanas ( *Amblyrhynchus cristatus* ), females reduce their investment in mate choice when food is scarce (Vitousek 2009). One hypothesis proposed to explain this pattern is that individuals in poor condition are more likely to accept less attractive partners (Jennions and Petrie 1997; Burley and Foster 2006). Building on this framework, we hypothesized that food availability would influence male mate choice. Specifically, we predicted that in starvation, males would reduce their preference bias for healthy females. In addition to influencing mate choice, parasitic challenges can also shape host behaviors known as infection‐avoidance behaviors, which reduce the risk of acquiring infections from conspecifics (Curtis 2014). Such behaviors, including the detection and avoidance of infected individuals, are thought to evolve under the strong selective pressures imposed by parasites (Parker et al. 2011). To distinguish between mate choice and infection‐avoidance behavior, we examined male preferences in non‐sexual social contexts. We predicted that males would show no preference for associating with healthy versus infected males and would be unable to discriminate between fungus‐infected and sterilized food.

## 2 Methods

### 2.1 Insects

*Tenebrio molitor* pupae were obtained from larvae breeding colonies that are reared at 27°C ± 1°C in the dark. The colony is fed with bran and corn meal (3:1) *ad libitum* and supplemented with apple pieces every other day. The food was sterilized (125°C ± 2°C for 15 min) to avoid any infection (Márquez‐García et al. 2016; Castro‐Vargas et al. 2017). For the experiments, we chose healthy adults, those without evident morphological defects (Rantala, Kortet, et al. 2003).

For the experiments, we selected virgin adult males and females that were 12 days post‐pupation. Insects were housed separately in well plates (Corning) starting from their pupal stage. We monitored the pupae daily to track the exact day they eclosed as adults. Once the adults reached 12 days of age, they were used in our experiments (Hernández‐Villanueva et al. 2023). Throughout their development, the insects were provided with food *ad libitum* and maintained under the same environmental conditions as those in the insectarium. Sample sizes for each experiment are provided in the results section.

### 2.2 Infection Status

**FIGURE 1:**
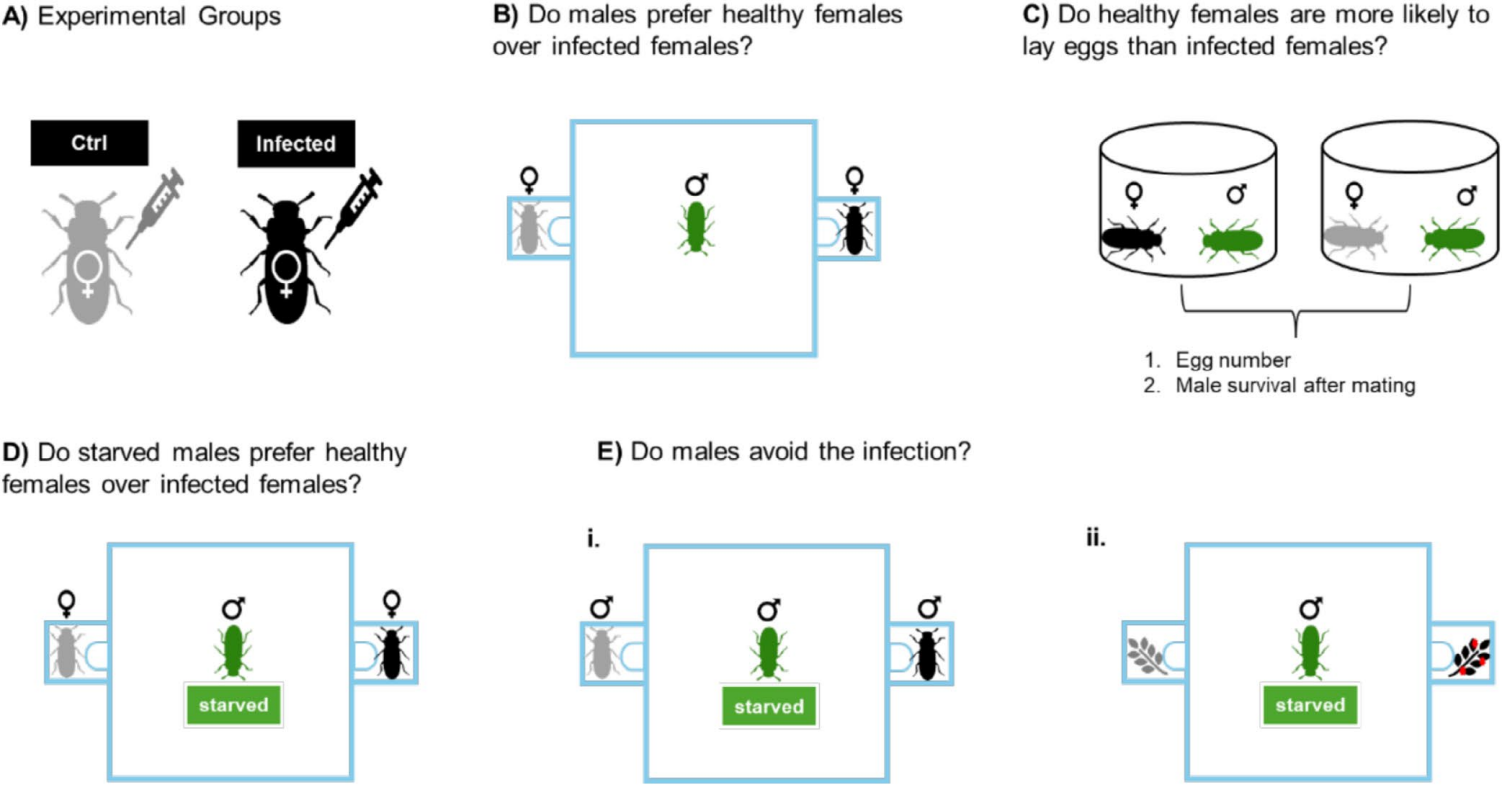
Experimental design. (A) Experimental groups were injected with either a control solution (Tween) or *Metarhizium anisopliae* spores suspended in Tween. (B) Experiment 1: A healthy focal male was placed in the center of an arena, with two females (one control, one infected) positioned in opposite chambers. (C) Experiment 2: Following mate choice trials, each female (control or infected) was paired with a healthy male in a 100 mL cage for 3 days to allow mating and oviposition. Post‐mating, male survival was recorded. (D) Experiment 3: Identical to Experiment 1, but focal males were food‐deprived for five days prior to testing. (E) Experiments 4 (i) and 5 (ii): In (i), a focal male was exposed to two males (one infected, one healthy) in a divided chamber. In (ii), the focal male was presented with two food sources (contaminated with *M. anisopliae* vs. sterilized control). In both cases, direct contact was prevented to assess preference, and males were food‐deprived to ensure motivation.

Once the insects reached 12 days of adult age, females of similar weight were selected and randomly assigned to one of two groups (Hernández‐Villanueva et al. 2023). Group 1 (control) received no infection, while group 2 was inoculated with a lethal dose (2 × 10^5^ spores/mL in Tween) of *Metarhizium anisopliae* (we confirmed that females die between 10 and 12 days after the three days of oviposition due to fungal infection). Males, also 12 days old, and females from each group were placed individually into separate wells of six‐well plates (Corning) with food provided. Females in group 1 were injected with 1 μL of tween (Merck) using a 10 μL syringe (Hamilton), whereas females in group 2 received 1 μL of *M. anisopliae* diluted in tween (Figure 1A). The infection was allowed to progress for 3 h before initiating mate choice experiments. Previous research suggests that, under similar pathogenic threats, females typically prefer healthy males over infected ones, or males may alter their signaling behavior as a form of terminal investment (Cordero‐Molina et al. 2024). Injection was used instead of topical application to ensure consistency in fungal exposure and to minimize variability in infection levels among individuals (Castro‐Vargas et al. 2017).

### 2.3 Behavioral Arena and Mate Preference

**FIGURE 2:**
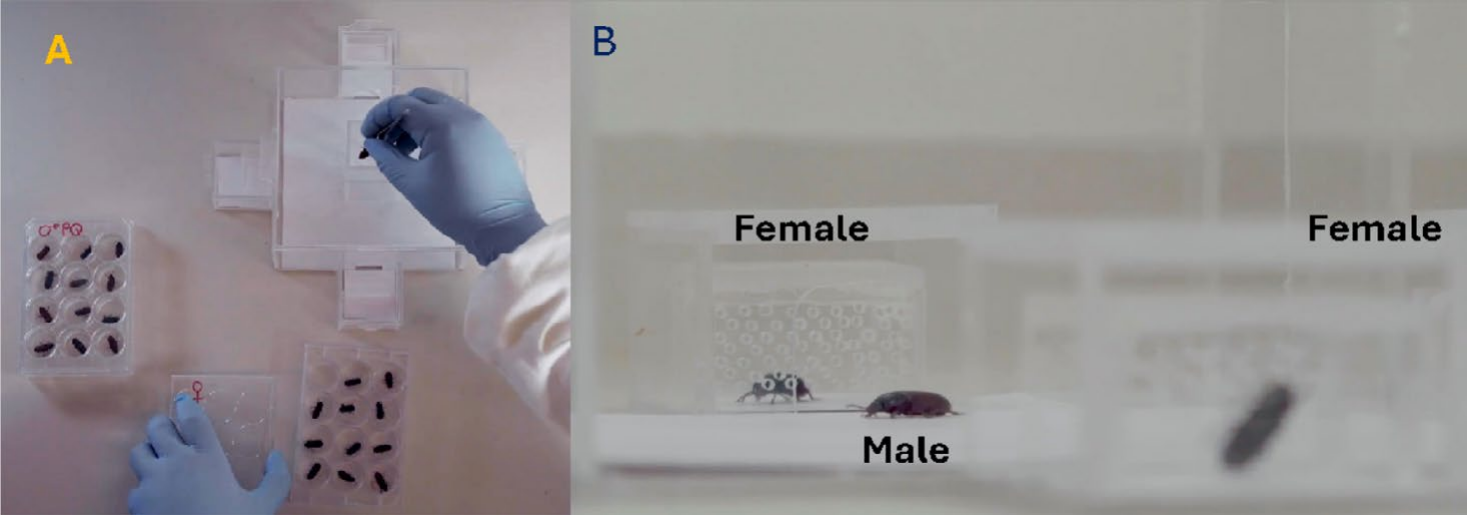
A behavioral arena (A) was used to test male choice. The behavioral arena consisted of a transparent acrylic box in which the choosing individual was placed, and opposing secondary chambers where the female mate options were located. Males chose between one of two female options, either infected or control (B).

The behavioral arena consisted of a transparent acrylic setup, with a main chamber (15 × 15 × 7.5 cm) in which the choosing individual was placed, and two opposing secondary chambers (4.5 × 4.5 × 2.5 cm) where the mate options (option 1 and option 2) were located (Figure 2A). Each secondary chamber contained a compartment designed to prevent physical contact between the choosier and the mate options, but with perforations allowing the exchange of chemical signals (Figure 2B). Clean filter paper was placed at the base of the arena as a substrate, which was replaced and the arena cleaned with 70% ethanol between each trial (Hernández‐Villanueva et al. 2023). Behavioral observations were conducted in darkness under dim red light (Hernández‐Villanueva et al. 2023). Before starting the observation, the sex of the choosier and the two mate options were placed in their respective chambers, and the setup was left to equilibrate for 5 min to allow habituation (Hernández‐Villanueva et al. 2023). During this period, the choosier individual was confined to the center of the main chamber by a removable device with holes to permit the flow of chemical signals. After 5 min, the focal individual was released and allowed to explore freely, visiting each of the secondary chambers containing the mate options for a period of 10 min (Hernández‐Villanueva et al. 2023; Cordero‐Molina et al. 2024). The time spent in each secondary chamber was recorded as the measure of mate choice (Ruiz‐Guzmán et al. 2021; Hernández‐Villanueva et al. 2023; Cordero‐Molina et al. 2024). Observations were carried out as described previously, with each trial conducted in a blinded manner to eliminate any potential observer bias.

### 2.4 Experiment 1. Do Males Prefer Healthy Females Over Infected Females?

At three hours post‐infection (Figure 1A), a focal male was placed at the center of the main chamber to assess mate choice between a healthy and an infected female within the behavioral arena, following the design previously described (Figure 1B). We recorded the time (in seconds) that males spent in the chamber of either the infected or the control female. Additionally, we evaluated whether males were more likely to visit the control female or the infected female first.

### 2.5 Experiment 2. Are Healthy Females More Likely to Lay Eggs Compared to Infected Females? And After Mating, Are Males That Mated With Infected Females More Likely to Die Than Males Mated With Healthy Females?

After the mate choice experiment, each female (control or infected) was paired with a healthy male that was kept separately in the Corning well plates. Each pair was housed in a 100 mL cage for 3 days to allow mating and oviposition. Ten days after the pairing, the percentage of females that successfully oviposited was recorded for each experimental group (Figure 1C).

After males mated, we compared the survival between those mated to infected or to healthy females. The mortality was assessed every day for 12 days (Figure 1C). Previous results in our laboratory have shown that 12 days is a good parameter to detect differences in survival after infection with the same fungus and the control groups (Medina‐Gómez et al. 2018; Contreras‐Garduño et al. 2019).

### 2.6 Experiment 3. Does Food Deprivation Affect Male Choice?

In this experiment, a focal male was introduced into the chamber and allowed to choose between two females: one infected with *2 ×* 10^5^ spores/mL and one healthy control female. Before the trial, focal males were food‐deprived for five days (Figure 1D). In the experiments of mate choice, we recorded the time the focal male spent in each female's compartment. In addition, we examined whether males were more likely to first visit the control female or the infected female.

### 2.7 Experiment 4 and 5. Do Males Avoid the Infection?

These assays consisted of two complementary experiments. In Experiment 4, a focal male was introduced into the main chamber, which contained two compartments, each housing a conspecific male: one infected with *2* × 10^5^ spores/mL and the other uninfected (Figure 2E,i). In Experiment 5, the focal male was presented with two food sources: one contaminated with *2* × 10^5^ spores/mL of fungus and the other sterilized as a control (Figure 2E,ii). As in Experiment 3, behavioral responses were quantified by recording the duration over 10 min, and we also examined whether males were more likely to first visit the control or the infected option.

## 3 Statistics

In our experimental design, each experimental treatment was compared with its respective control. As the data met the assumptions of normality and homogeneity of variances, the time males spent in front of females was analyzed using paired *t*‐tests, with results reported as mean ± standard error. The probability of egg production was assessed using a chi‐square test, based on the likelihood of females laying eggs within their respective groups. Survival curves between treatments were compared using log‐rank tests. All statistical analyses were conducted in SPSS Statistics version 22.0.0.0.

## 4 Results

**FIGURE 3:**
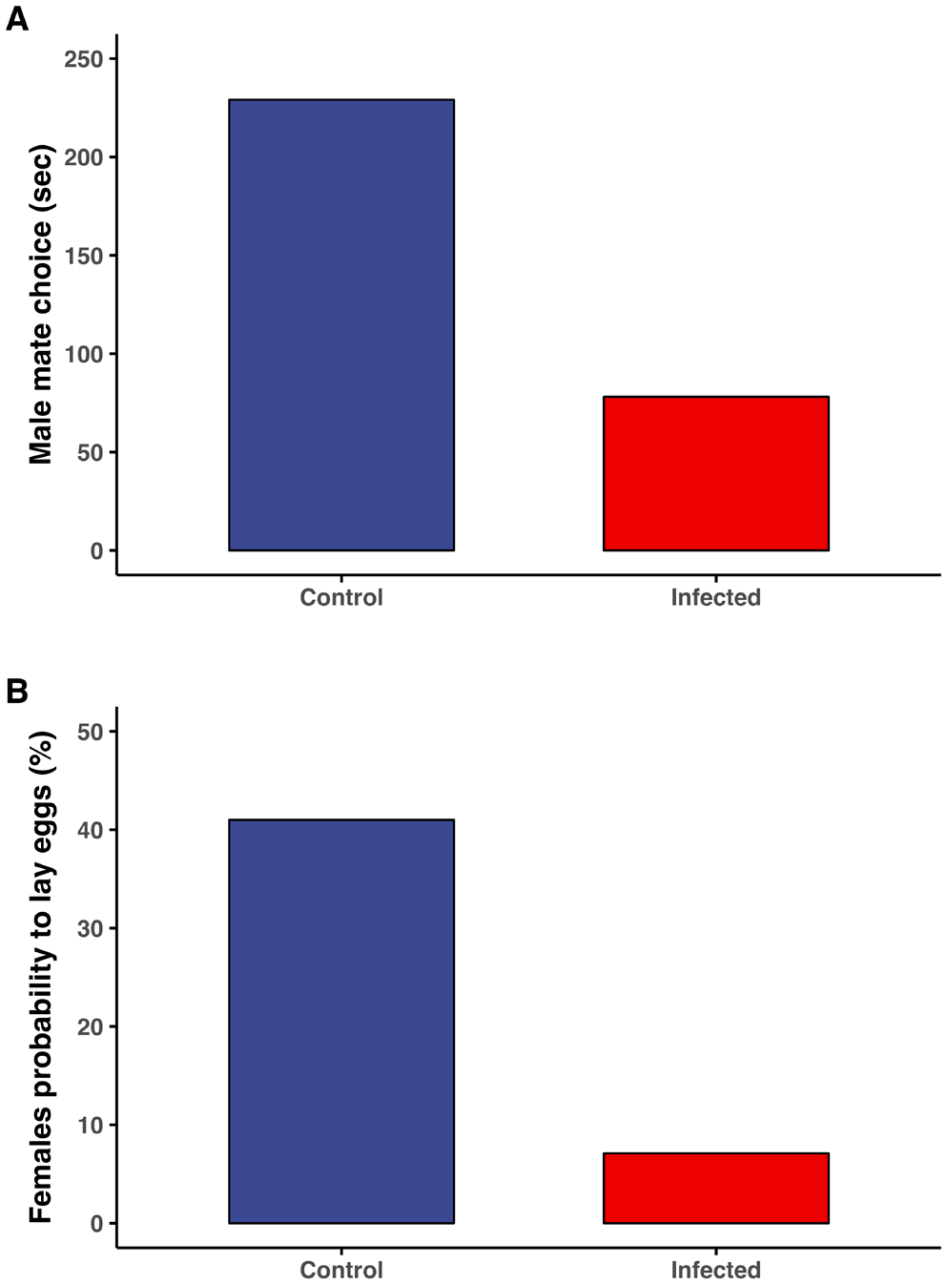
Males preferred the healthy females over those infected with the fungus (A). The male preference is shown as the time (secs) that males spend with each female option. The figure also shows that it was more likely that healthy females lay eggs than the infected females (B).

In the first mate choice experiment, we assessed whether males exhibited mate choice. Males spent significantly more time with healthy females (229.07 ± 36.08 s, *n* = 31) compared to parasitized females (78.09 ± 19.83 s, *n* = 31; paired *t*‐test = 3.1, df = 30, *p* = 0.004; Figure 3A). Males were not more likely to visit first the control females (14 out of 30) than the infected (16 out of 30) females (*χ* ^2^ = 0.13, df = 1, *p* = 0.71).

Results from the second experiment revealed that parasitized females were less likely to oviposit (7.1%) compared to healthy females (41%; *χ* ^2^ = 12.5, df = 1, *p* = 0.0004; Figure 3B). Additionally, male survival did not differ between those mating with healthy versus parasitized females. Males that mated and remained in the same cage for three days with the control or infected females showed no difference in survival for the other 12 days (log‐rank test: *χ* ^2^ = 0.005, df = 1, *p* = 0.94). Thus, fungal infection in females does not appear to impose a direct survival cost on males.

The third experiment revealed that starved males did not prefer healthy (94.9 ± 17.87 s, *n* = 30) over parasitized females (86.2 ± 21.58 s, *n* = 30; paired t‐test = 0.26, df = 29, *p* = 0.79). Males were not more likely to visit the control first (12 out of 30) than females over the infected (18 out of 30) ones (*χ* ^2^ = 1.2, df = 1, *p* = 0.27).

In experiment 4, when starved males were given the choice between parasitized and non‐parasitized control males, no significant preference for females of either type was observed between the two groups (Control: 90.7 ± 21.1 s, *n* = 31; Infected: 100.13 ± 21.32 s, *n* = 31; paired *t*‐test = −0.15, df = 29, *p* = 0.87). Males were not more likely to visit the control first (16 out of 30) than females over the infected (14 out of 30) ones (*χ* ^2^ = 0.13, df = 1, *p* = 0.71). Similarly, in Experiment 5, when starved males had the option of choosing between sterilized food (96.92 ± 39.07 s, *n* = 13) and food infected with the fungus (87.08 ± 20.08 s, *n* = 13), males consumed both types of food in similar proportions (paired *t*‐test = −0.2, df = 12, *p* = 0.84). Finally, we did not observe that males visited the control food (17 out of 30) over the infected food (13 out of 30) (*χ* ^2^ = 0.53, df = 1, *p* = 0.46).

## 5 Discussion

Our results suggest that male *T. molitor* prefer healthy females over parasitized ones, and this preference aligned with female reproductive output. Healthy females were substantially more likely to oviposit than parasitized females, suggesting that males may increase their reproductive success by preferentially associating with mates in better condition. This finding supports the hypothesis that male mate choice can enhance fitness by biasing reproductive investment toward higher‐quality partners. Male choice appears to be shaped by indirect benefits in terms of the higher reproductive potential of healthy females. This is consistent with the idea that males, like females, can benefit from discriminating among potential mates when such discrimination influences reproductive success (Kokko and Johnstone 2002; Edward and Chapman 2011; Ah‐King and Ahnesjö 2013).

Interestingly, male mate choice was a condition‐dependent behavior. Under starvation, males did not discriminate between healthy and parasitized females, nor did they avoid fungus‐infected food or infected conspecific males. This suggests that food deprivation reduces mate choosiness in *T. molitor* , likely because the energetic constraints associated with starvation reduce choosiness. The evidence suggests that costs reduce choosiness (Jennions and Petrie 1997). Hence, males are more likely to be choosy if energetic costs affect their mating opportunities (Wong and Jennions 2003). For example, in *Pseudomugil signifer* , the costs of swimming, and hence, the metabolic cost, reduced the males' choosiness to court the more fecund females (Wong and Jennions 2003). The condition‐dependent choice has been proposed in different species (Jennions and Petrie 1997; Burley and Foster 2006), and our results support the hypothesis that environmental stressors can lower discrimination thresholds. In resource‐rich environments, males may afford to be more selective, whereas under stress, they may accept mates of lower quality.

The preference for healthy females may be explained as honest signals of condition, or as an active avoidance of pathogen transmission. Our results revealed that mating with parasitized females did not impose survival costs on males due to the transition of infection. This suggests that male mate choice in this system is not primarily driven by direct avoidance of infection risk. The lack of avoidance in the male–male association (experiment 4) and the lack of preference for consuming infected versus sterile food (experiment 5) reinforce our interpretation. Since fungal infection alters host condition and potentially affects chemical cues, males may rely on these changes to assess female quality. Previous work in *T. molitor* and other insects shows that parasitism and stress can alter cuticular hydrocarbons and pheromonal signals (Hurd and Parry 1991; Rantala et al. 2002; Rantala, Kortet, et al. 2003; Rantala, Vainikka, et al. 2003; Vanderwel et al. 2017; Ruiz‐Guzmán et al. 2021). Our results extend this evidence by showing that males respond behaviorally to infection‐related changes in females, even in the absence of direct infection risk. Taken together, these findings suggest that both sexes of *T. molitor* are capable of discriminating between healthy and parasitized partners. Such mutual mate choice has been previously documented in *T. molitor* under oxidative stress (Ruiz‐Guzmán et al. 2021) and suggests that accurate assessment of partner condition is crucial for maximizing reproductive success in this species. So, given that parasitized females are less fecund, male preference for healthier females seems to be adaptive, suggesting that male mate choice in *T. molitor* is based on the honest signals of condition. Mutual mate choice is present in other invertebrates. For example, in the scorpion *Urophonius achalensis*, there is a mutual mate choice influenced by their own quality and that of their partner (Oviedo‐Diego et al. 2025). Hence, mate choice in males and females can be shaped by ecological conditions, individual state, and the costs and benefits of discrimination.

The fact that male choice is relaxed under starvation highlights the importance of considering environmental and physiological context when evaluating sexual selection dynamics. Our results also support the hypothesis of the preference for honest signals of condition rather than an active skew derived from avoidance of parasite transmission. Future work should explore the mechanistic basis of how males perceive female infection status, including the role of chemical communication, and test whether these patterns generalize across other insect species.

## Author Contributions

**Carolina Castillo:** investigation (equal), methodology (equal), writing – original draft (equal), writing – review and editing (equal). **Jimena Meneses Placencia:** investigation (equal), methodology (equal), writing – original draft (equal), writing – review and editing (equal). **Judith Ulloa:** investigation (equal), methodology (equal), writing – original draft (equal), writing – review and editing (equal). **Sagrario Cordero‐Molina:** formal analysis (equal), writing – original draft (equal), writing – review and editing (equal). **Constantino Macías García:** conceptualization (equal), writing – original draft (equal), writing – review and editing (equal). **Indrikis Krams:** conceptualization (equal), funding acquisition (equal), writing – original draft (equal), writing – review and editing (equal). **Jorge Contreras‐Garduño:** conceptualization (equal), funding acquisition (lead), project administration (lead), supervision (lead), writing – original draft (lead), writing – review and editing (lead).

## Ethics Statement

The authors have nothing to report.

## Conflicts of Interest

The authors declare no conflicts of interest.

## Supporting information


**Data S1:** ece372178‐sup‐0001‐Supinfo.xlsx.

## Data Availability

The data is provided as Supporting Information.
